# Predicting *Zea mays* Flowering Time, Yield, and Kernel Dimensions by Analyzing Aerial Images

**DOI:** 10.3389/fpls.2019.01251

**Published:** 2019-10-11

**Authors:** Guosheng Wu, Nathan D. Miller, Natalia de Leon, Shawn M. Kaeppler, Edgar P. Spalding

**Affiliations:** ^1^Department of Botany, University of Wisconsin–Madison, Madison, WI, United States; ^2^Department of Agronomy, University of Wisconsin–Madison, Madison, WI, United States

**Keywords:** normalized difference vegetation index, *Zea mays*, unmanned aerial vehicle, flowering time, grain yield, kernel dimension

## Abstract

Image analysis methods for measuring crop phenotypes may replace traditional measurements if they more efficiently and reliably capture similar or superior information. This study used a recreational-grade unmanned aerial vehicle carrying a spectrally-modified consumer-grade camera to collect images in which each pixel value is a vegetation index based on the normalized difference between the blue and near infrared wavelength bands (BNDVI). The subjects of the study were *Zea mays* hybrids with good yield potential grown in 4-row plots. Flights were conducted at least once per week during three successive growing seasons in south-central Wisconsin. Average BNDVI for each plot (genotype) rose steadily through June, peaked in July, and then declined as plants matured. BNDVI histograms changed shape over the season as the canopy concealed soil, became more uniformly green, then senesced. Principal Components Analysis (PCA) captured the change in histogram shape. PC1 represented canopy closure. PC2 represented the mean of the BNDVI distribution. PC3 represented the spread of the distribution. Correlation analysis showed that flowering time correlated with PC2 and PC3 best (r ≈ 0.5) a few days before the event (day in which 50% of the plants exhibited tassels). Three ears were picked from each plot to quantify kernel dimensions by image analysis before each plot was mechanically harvested to determine grain weight per plot. Correlations between this measurement of yield and PC2 were low in June but exceeded 0.4 within 10 days after flowering. Kernel length correlated similarly with PC2. The correlation between PC2 and kernel thickness displayed a similar but inverted time course. These results indicate that greater mid-season BNDVI values correlate positively with yield comprised of tall, thin kernels. Partial least squares regression performed on the BNDVI time courses predicted flowering time (r = 0.54–0.79) and yield (r = 0.4–0.69). This three-year experiment demonstrated that readily available hardware and software can create a phenotyping platform capable of predicting maize flowering time, yield, and kernel dimensions to a useful degree.

## Introduction

Genotype-to-phenotype studies will produce stronger conclusions and the process of improving quantitative traits will accelerate if the methods for measuring crop plant phenotypes are as discriminating as the next-generation DNA sequencing methods used to characterize the genotypes (Edwards et al., 2013; Bevan et al., 2017). Image analysis methods have the potential to provide the needed phenotype data. When successfully applied, they measure standard traits more precisely, objectively, and automatically than manual methods, and they can measure informative features for which there is no manual equivalent. However, acquiring image data from which useful phenotype data can be extracted is more challenging in a field experiment than in situations where the imaging scene can be controlled and optimized. White et al. (2012) and Araus and Cairns (2014) discuss the many challenges to field-based phenotype measurements using cameras and other sensors.

Satellites and piloted aircrafts have been used to obtain aerial images of field-grown crops for various applications. For instance, Landsat and Sentinel-2 satellites collect red and near-infrared (NIR) wavelength bands from regularly revisited locations to assess global vegetation and crop health (Rouse et al., 1973; Tucker and Choudhury, 1987). Unfortunately, the spatial resolutions of these methods (Issei et al., 2010) are on the order of meters or tens of meters, which is not fine enough to serve the phenotype measurement needs of many crop plant research projects. Cameras and other sensors mounted to ground-based vehicles, overhead gantries, or cable supports can collect detailed phenotype information (Furbank and Tester, 2011; Montes et al., 2011; White et al., 2012; Deery et al., 2014). Growing in popularity is the small unmanned aerial vehicle (UAV) programmed to fly along a path defined by a series of waypoints marked by global positioning system (GPS) coordinates (Colomina and Molina, 2014; Yang et al., 2017). When outfitted with cameras, UAV technology provides a low-cost approach to collecting images with the required spatial and temporal resolutions. Potential uses of UAV technology include measuring seedling emergence, plant height, ground canopy cover, leaf angle distribution, leaf area index, and overall crop health in various crop species (for a review, see Yang et al., 2017). For example, a surface model generated from aerial color images measured barley and rice crop height (Bendig et al., 2013a; Bendig et al., 2013b), height and growth rate in maize and wheat (Holman et al., 2016; Li et al., 2016), and sorghum and maize plant height at many points across the growing season (Pugh et al., 2018). UAV platforms were used to measure vegetation indices based on ratios of spectral bands captured by a camera in tens of thousands of wheat plots (Haghighattalab et al., 2016). Hyperspectral sensors mounted on UAVs collected many wavelength bands from which the biomass and nitrogen content of wheat, barley, and maize were estimated (Montes et al., 2011; Pölönen et al., 2013). A UAV platform carrying a hyperspectral imager and a thermal camera was used to detect water stress in a citrus orchard (Zarco-Tejada et al., 2012). Numerous studies also used UAV platforms to measure maize plant lodging (Chu et al., 2017), ground canopy cover in wheat (Yu et al., 2017), plant density, early vigor, and radiation interception in maize (Liebisch et al., 2015), as well as soil and plant interactions, and weed management (Shi et al., 2016).

A desirable application of UAV technology is to predict crop yield based on a vegetation index derived from aerial images. A common vegetation index is the normalized difference vegetation index (NDVI). It is based on the principal that chlorophyll absorbs red and blue wavelengths while reflecting near-infrared radiation (Tucker, 1979). To measure NDVI, the camera’s detector must be sensitive to near-infrared (NIR) and blue or red wavelengths. NDVI is calculated at each pixel according to Equation 1.

(1) $$\mathrm{NDVI}=\frac{\left[\mathrm{NIR}-\mathrm{Red}\right]}{\left[\mathrm{NIR}+\mathrm{Red}\right]}$$

A value of 1 indicates green plant material due to absorption of red wavelengths relative to the NIR reference, while -1 indicates the lack of red-absorbing material. Higher NDVI values usually indicate greater vigor, and therefore may relate to yield potential and abiotic/biotic stress tolerance (Candiago et al., 2015; Condorelli et al., 2018). For example, a UAV-based platform generated NDVI images that were used to estimate the effects of low nitrogen stress on maize yield (Zaman-Allah et al., 2015). Makanza et al. (2018) used RGB image data to create a novel senescence index. It showed a moderately high heritability and a strong genetic correlation with maize grain yield. Cerrudo et al. (2017) and Condorelli et al. (2018) used NDVI and other spectral-based indices to study the effects of heat and drought stress on grain yield in maize and wheat. In rice, dynamic changes in vegetation indices during early to middle growth stages were used to predict rice grain yield (Zhang et al., 2019). For wheat, a fairly high correlation of vegetation index values with yield was observed (Guan et al., 2019).

Many studies employing UAVs to measure a vegetation index focused on growth, phenology, and stress responses of crops (Yang et al., 2017). The differences between comparison groups in such studies are often large. Fewer studies have used a UAV imaging platform to discern subtle differences among genotypes experiencing a similar environment, which a tool must achieve if it is to be useful in a breeding program. Liebisch et al. (2015) and Han et al. (2018) included multiple maize genotypes in their analyses of NDVI acquired from airborne platforms but testing for differences between them was not a feature of their reports. We designed the present study to determine if analyses of vegetative index values could discern differences in flowering time, grain yield, and kernel dimensions between different hybrid maize plants, each with good yield potential.

## Materials and Methods

### Germplasm and Field Trials

The field experiments were conducted between June and October of 2016, 2017, and 2018 at the University of Wisconsin’s Arlington Agricultural Research Station in Arlington, Wisconsin, USA (43.33° latitude and -89.34° longitude). Twenty-five F1 maize hybrids were planted as randomized complete block designs on 5/24/2016, 5/10/2017, and 5/24/2018, then harvested in late-October. Most but not all of the genotypes were used each year. The complete list is presented in **Supplementary Table S1**. Each replicated plot (i.e., two trials) contained 4 rows with 40 plants each. The rows were 4.57 m long, 0.76 m apart, and the target density was 78,200 plants ha^−1^.

### Flowering Time

Flowering time is typically scored as the date when 50% of the plants in a plot display tassels for the male, and when 50% of the ears have extruded silks to receive pollen. These standard measures of flowering time were made by repeated visual inspection of each plot during the flowering period each year. Specifically, to record flowering data, we visited each plot every other day during the flowering period in late July. We recorded the day when half of the plants in a plot were shedding pollen or displayed silks, for each genotype.

### Kernel Dimensions

To measure kernel dimensions, three mature ears were hand-picked from each plot prior to machine harvest. We used a previously developed image analysis pipeline (Miller et al., 2017) to measure kernel traits for each of the genotypes for each year.

### Grain Yield

A combine harvester measured the mass of grain and percent moisture for each plot at the end of growing season (mid-October). Grain yield, which includes the grain harvested by the combine and the hand-picked ears, was adjusted to 15.5% grain moisture. Grain yields for each genotype for each year are shown in **Supplementary Table S1**.

### Unmanned Aerial Vehicle Platform

The UAV platform consisted of a quadcopter (model IRIS+; 3D Robotics, Berkeley, CA, USA) and a Canon S110 compact camera attached to a gimbal that maintained the camera in the nadir position. The sensor in the camera was modified by Llewellyn Data Processing (http://maxmax.com) such that the red channel sensed wavelengths between 670 and 770 nm, with a peak at approximately 710 nm. Therefore, instead of a red, green, and blue (RGB) image, this camera created an NIR, green, blue (NGB) image. A plot of the spectral sensitivity of the modified sensor provided by Llewellyn Data Processing is presented in **Supplemental Figure S1**. Custom software controlled the camera to enable time-lapse imaging at a frequency of approximately one frame per second. The focal length was 5 mm, the aperture was f/2.5, the ISO sensitivity was 200, the shutter speed was 1/1600 s, and the image size was 3000 X 4000 pixels. The files were saved in Joint Photographic Expert Group (jpeg) format. The ground sample distance of the full-field image was 0.7 cm per pixel. We used Mission Planner autopilot software (http://www.arducopter.co.uk/advanced.html) to design a flight route. The flight altitude was 25 m and the flight speed was 1 m s^-1^. These specifications combined to produce images that overlapped at least 60% forward and laterally. Flights were conducted only during fully clear (cloudless) periods between 10 a.m. and 3 p.m. at least once per week during each of the three seasons, with a higher frequency around the flowering period. Early season flights were not performed in 2018 because 2016 and 2017 results indicated June time points were not sufficiently useful to justify the analyses. In late August 2018, a severe storm caused widespread lodging so late season flights were not performed.

### Image Processing and BNDVI Extraction

Agisoft PhotoScan Professional software (www.agisoft.com) was used to construct a full-field image from the individual frames after each flight. The parameter settings, including raw image alignment, mesh building, and orthoimage generation, were held constant for each field image construction. The resulting three channel NGB image of the field was then converted into a blue channel-based NDVI layer (BNDVI, Equation 2) by a plugin for ImageJ/FIJI that can be downloaded here: https://github.com/nedhorning/PhotoMonitoringPlugin/tree/master/downloads.

(2) $$\mathrm{BNDVI}=\frac{\left[\mathrm{NIR}-\mathrm{Blue}\right]}{\left[\mathrm{NIR}+\mathrm{Blue}\right]}$$

**Figure 1:**
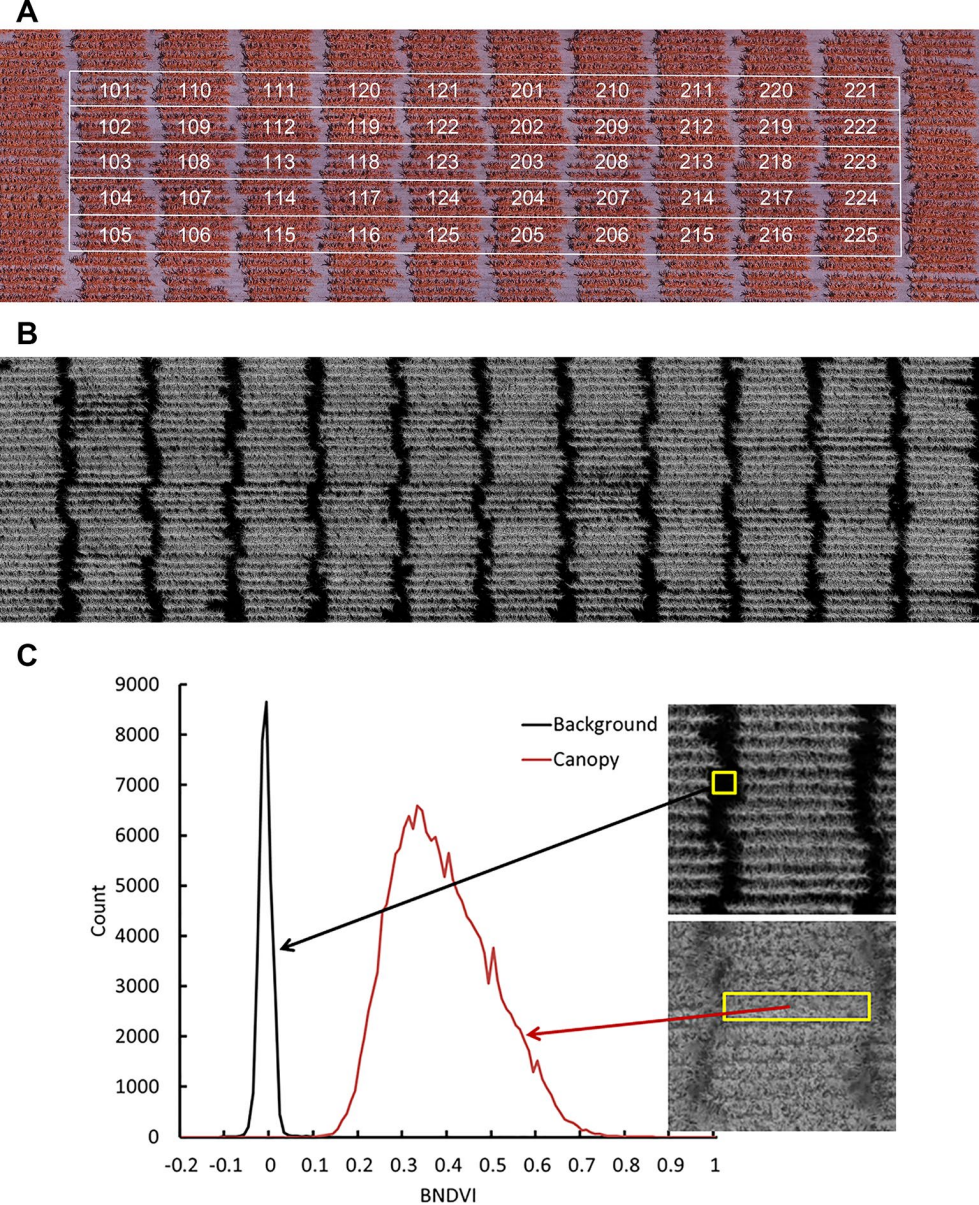
Aerial image data obtained by the UAV platform. **(A)** A representative image of a field containing all the genotypes obtained with a camera modified to have an NIR, green, and blue channel. **(B)** The calculated BNDVI image layer. **(C)** Histogram of BNDVI values from canopy (red line) and soil background (black line). The threshold 0.06 was used as cutoff to extract plant BNDVI in all plots.

**Figure 1A** shows a representative NGB image and **Figure 1B** shows the corresponding BNDVI image. Each pixel in the BNDVI image is associated with a value ranging from -1 to 1. **Figure 1C** uses histogram representations of images within regions of interest to demonstrate that pixels corresponding to ground or maize canopy are reasonably well separated by choosing 0.06 as the threshold (background < 0.06 < plant).

Individual 4-row plots were manually cropped from the full-field BNDVI image. The alley separating ends of rows was excluded (**Figure 1**). Each plot sub-image consisted of approximately 2 x 10^5^ pixels, each representing a BNDVI value ranging from -1 to 1. The average BNDVI values for each genotype at the time of flowering for each year are presented in **Supplementary Table S1**. Most analyses were performed not on average BNDVI values but on the histogram of BNDVI values for each plot at each flight date. These histograms were treated as quantitative, time-dependent phenotypes to be compared between genotypes.

### Principal Component Analysis (PCA)

The BNDVI values between 0 and 1 were placed into 999 equally spaced bins to create a histogram for each four-row plot. The histograms from all genotypes (*g*), trials (t = 2), flight-dates (d), and years (y) were stacked into the matrix X with *n* = × *t* × *d* × *y* rows and *m* = 999 columns to make a *n* × *m* matrix containing one histogram for every plot measured during the study. Principal component analysis reduced the number of variables and provided a potentially interpretable set of latent scores based on the eigenvectors of the covariance matrix of X, or the right singular vectors from the singular value decomposition of the column-mean centered X. The cumulative sum of the eigenvalues of the covariance matrix of X divided by the total variance is the percent variance explained. We took the first 15 components, which explained greater than 99% of the variance and created three matrices (X_2016_, X_2017_, X_2018_), each with *g* × *t* × *d* rows and 15 columns, which contained the scores used for partial least squares regression analysis of the traits.

### Partial Least Squares Regression (PLS-R)

To prepare for partial least squares regression (PLS-R), a technique commonly used when the number of observations is greater than the number of trials, the measured traits (*r* = tassel time, silk time, kernel thickness, kernel width, kernel depth, and grain yield) for all genotypes (*g*) were stacked into three matrices (Y_2016_, Y_2017_, Y_2018_) with 2*g* rows (because there were two trials of each genotype) and one column for each separate *r*. The goal of the PLS-R was to obtain a low dimensional model that maximized the covariance between X_year_ and Y_year_. For each X_year_ and Y_year_ pair, one-hundred PLS-R models were constructed, with 30% of the data randomly selected for hold out. To guard against overfitting, the model was applied to the hold-out group and the factor threshold (f_T_) was found where the predictive power of the model began to diminish. The average threshold (f_A_) across the 100 hold-out draws was computed and reported in **Table 1** along with the correlation coefficients that indicate the degree of prediction accuracy. We also performed the PLS-R using the original histogram values instead of their corresponding 15 PCs. The predictions were generally slightly higher when the original histogram data was used but the distribution of correlation values obtained from 100 different hold-out trials were not statistically different (p = 0.05).

**Table 1:** Correlation coefficients (r) of partial least squares regression analysis of various maize traits. Numbers in parentheses are the minimum number of factors needed for accurate predictions.

Year	Tassel	Silk	Grain yield	Kernel length	Kernel thickness
2016	0.79 (4)	0.75 (6)	0.69 (4)	0.44 (5)	0.31 (4)
2017	0.54 (6)	0.55 (7)	0.45 (4)	0.41 (8)	0.35 (5)
2018	0.77 (6)	0.76 (7)	0.40 (3)	0.37 (4)	0.39 (7)

## Results

### BNDVI Profiles During Growing Season

**Figure 2:**
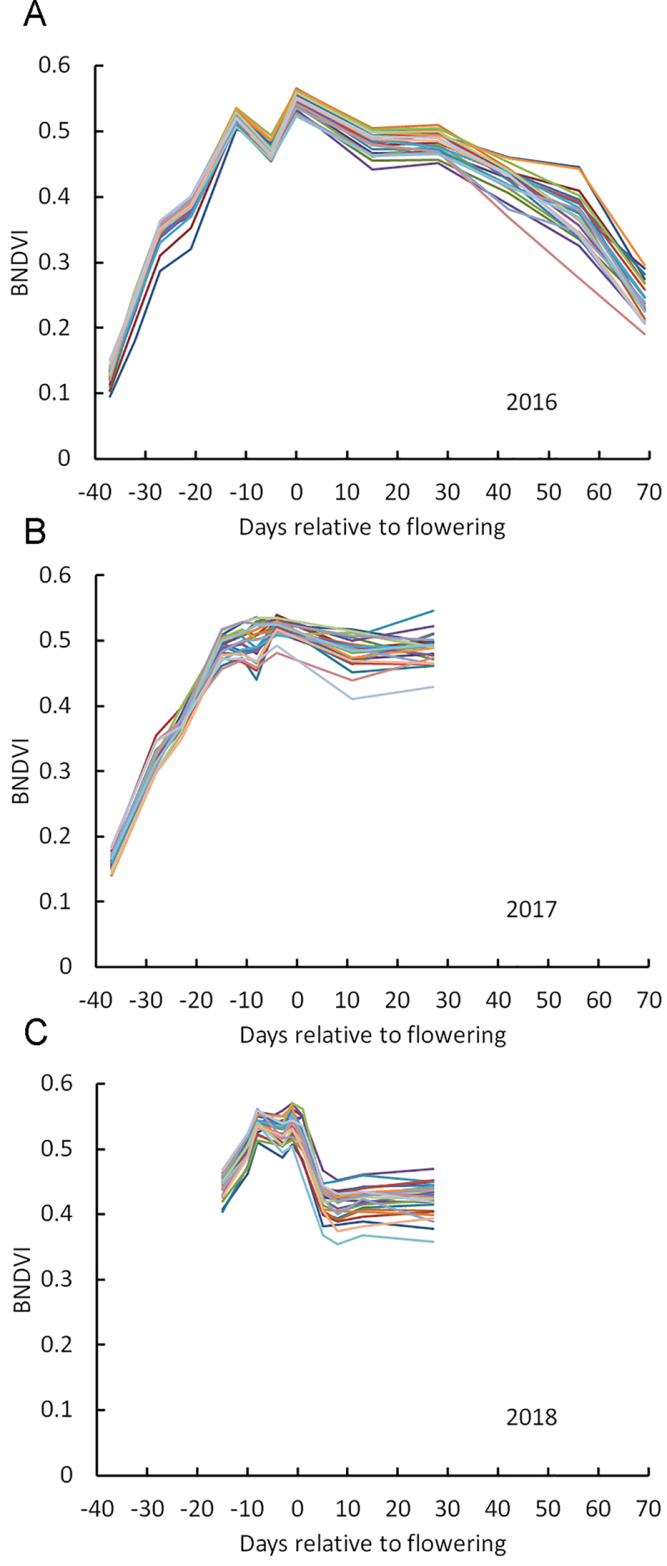
Change in BNDVI for different maize genotypes across the growing season. **(A)** 2016, **(B)** 2017, and **(C)** 2018. Two vertical lines in each panel mark the flowering time range for all genotypes.

**Figure 2A** shows mean BNDVI values for the canopy pixels (average of the duplicated plots) for each of the 25 hybrids grown in 2016, at each flight date. BNDVI increased during the early growing season, peaked during the flowering period, and then decreased as senescence progressed as previously reported (Viña et al., 2004). A dip in BNDVI was consistently observed a few days preceding flowering for all genotypes in 2016. This phenomenon was observed again in 2017 and 2018 although it was not as obvious as in 2016 (**Figures 2B, C**). Han et al. (2018) also detected a transient reduction in NDVI near flowering time. The appearance of tassels in the images at flight dates corresponding to the dip support the idea that the reduced BNDVI was due to a small but significant contribution of non-green tassel material entering the scene. These results indicate that BNDVI could be monitored to detect flowering time.

### Principal Components Analysis of BNDVI Histograms

**Figure 3:**
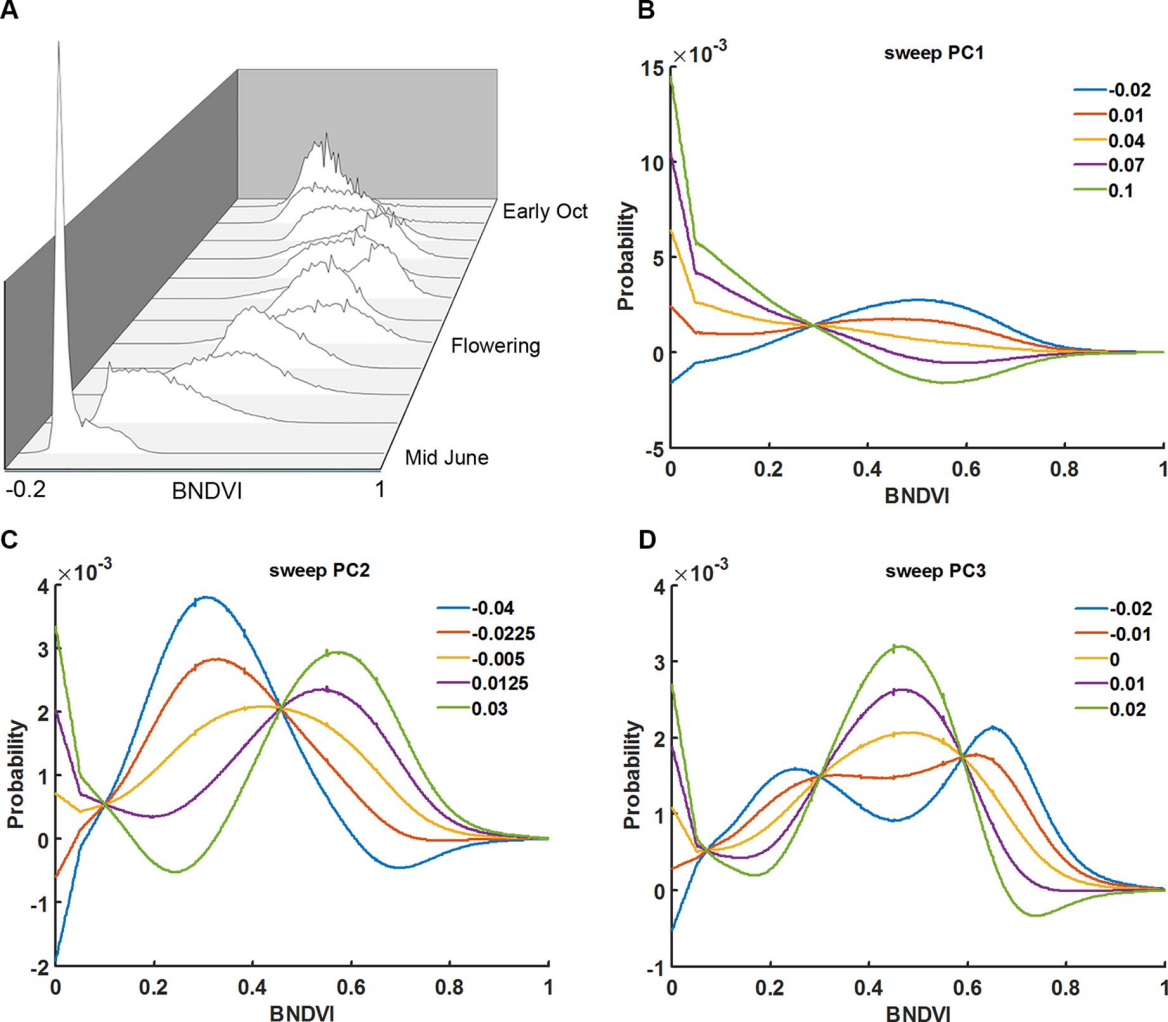
Principal component analysis of vegetation index histograms. **(A)** BNDVI histograms from a representative genotype shifted during the 2016 growing season. Parameter sweeps of the back-projection models for the three eigenvectors (PCs) that explain 96% of the variance in histogram data from all trials, years, genotypes, and flight dates **(B–D)**. PC scores ranged from the minimum score to the maximum score at five steps along each eigenvector. See the Results section for an interpretation of the effects the three PCs.

Instead of calculating the mean BNDVI value for each plot, at each flight date, the BNDVI values were treated as a frequency histogram. **Figure 3A** shows histograms of BNDVI values including those below the 0.06 threshold from one of the plots at each flight date during the 2016 season. The distributions change shape as the canopy covers the ground, becomes greener, then senesces.

The shape of histograms may carry important information that aids the prediction of maize traits. The meaning of the eigenvectors was explored by independently sweeping the score values, back-projecting the corresponding histogram model, and adding back the average. Shown in **Figures 3B–D** are the back-projection models for the three eigenvectors that explain 96% of the variance. The first principal component (PC1) accounts for 71% of the total variability. The sweeps indicate that it captures the shift in the distribution of BNDVI from low values corresponding to soil to high values indicative of maize canopy. PC2, which accounts for 19% of the total variance, shifts the overall distribution to greater BNDVI values. PC3, which explains 6% of the variance, changes the shape of histogram toward a bimodal distribution.

**Figure 4:**
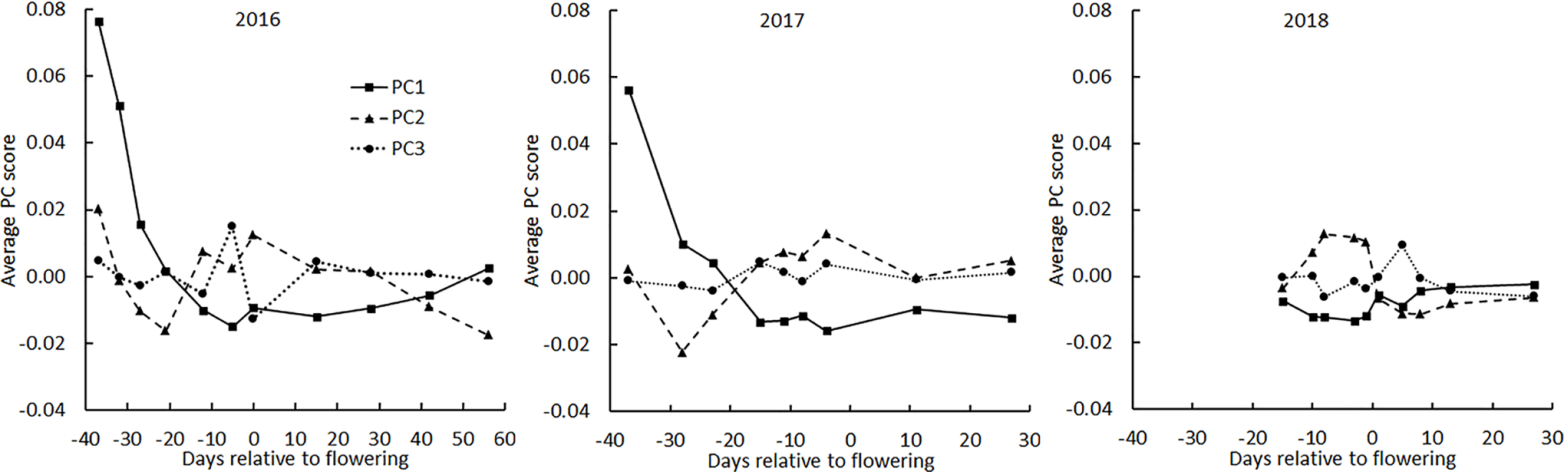
Average principal component scores derived from BNDVI histograms of all genotypes during the three growing seasons. The average date of flowering of all genotypes was set as day 0.

The principal component scores were plotted as a function of flight date made relative to the mean flowering date for all genotypes for each of the three years (**Figure 4**). In 2016, the year in which flights were spread most evenly across the season, PC1 rapidly decreased early, consistent with it interpreting soil background. The same was observed in 2017 but in 2018 the early season was not surveyed. In each year, PC2 showed a tendency to drop from a mid-season high, consistent with it interpreting average greenness. No pattern in PC3 across the season was apparent, consistent with PC3 representing only 6% of the total variance.

**Figure 5:**
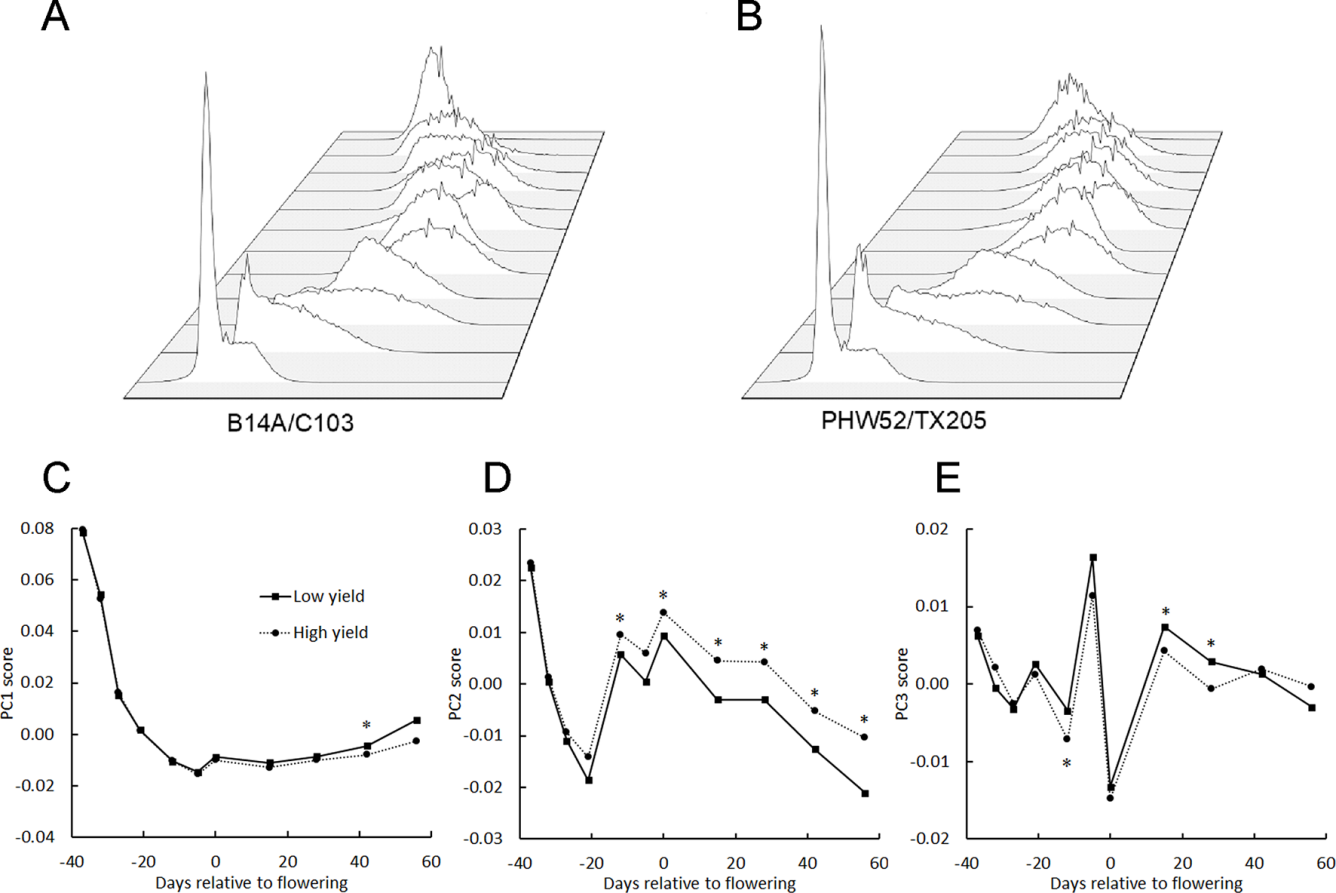
BNDVI histogram principal components change across the growing season. BNDVI histograms from a low yield **(A)** and a high yield **(B)** genotype change shape during the 2016 growing season. **(C–E)** The principal components of the histograms corresponding to the 6 lowest-yielding genotypes (Low yield) and the 7 highest-yielding genotypes (High yield) in 2016 were averaged and plotted across the growing season. Significant differences in PC1 **(C)**, especially PC2 **(D)**, and PC3 **(E)** were found between the low and high yielding pools of genotypes. An asterisk above a point indicates P < 0.05.

The main aim of this study was to find factors that could be used to distinguish important traits among maize genotypes. **Figures 5A, B** show the histograms from a low yield genotype (B14A/C103; 10.1 kg/plot) and a high yield genotype (PHW52/TX205; 27.6 kg/plot) at each flight date in 2016. **Figures 5C-E** show the seasonal course of average PC1, PC2, and PC3 from 6 genotypes with lowest grain yield and 7 genotypes with highest grain yield in 2016. There was no difference in PC1 between the two genotype groups before the flowering period but 30 days after flowering, PC1 for the low yielding genotypes rose more quickly than the high yielding genotypes. PC2 was higher for the high-yielding genotypes for most of the season, while PC3 did not demonstrate any obvious trend across the whole season. The PC scores of these two contrasting genotypes indicate that higher yield is associated with a more persistent and full green canopy after flowering. This comparison of genotypes contrasting with respect to plot yield gave reason to seek other correlations between the BNDVI histogram principal components and other agronomically relevant traits.

### Flowering Time: Correlations With Histogram Principal Components

**Figure 6:**
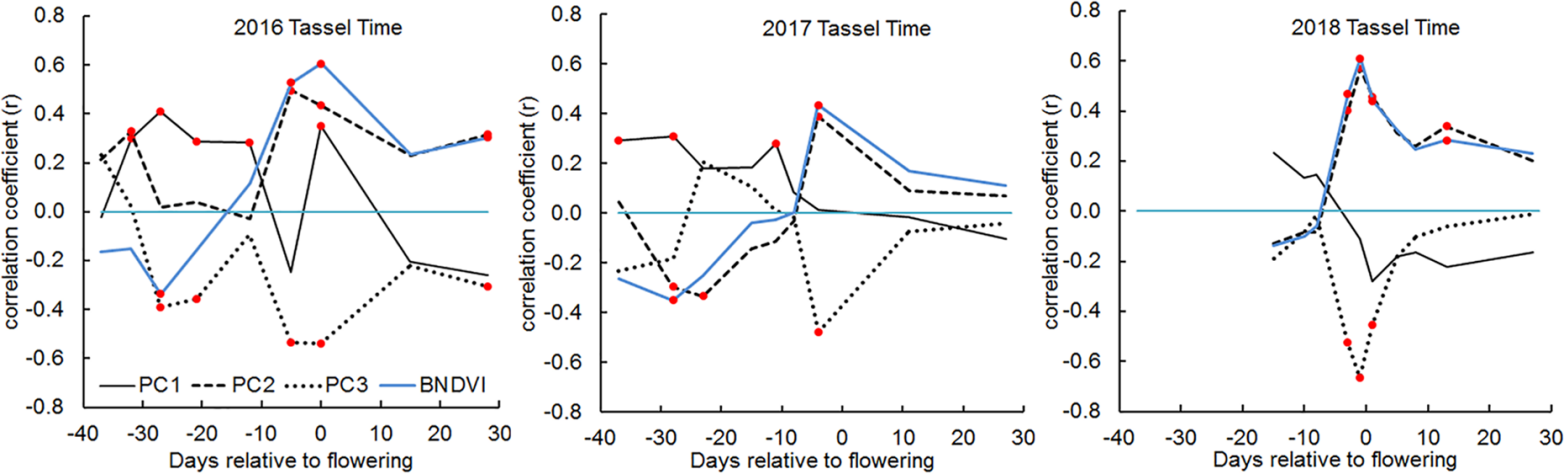
Correlations between male flowering (tassel) time and BNDVI histogram principal components in 2016, 2017, and 2018. In each panel, the blue line indicates the correlation between tassel time and mean BNDVI. The other lines represent correlation with the indicated principal component. Any correlation value that is significant at the P < 0.05 level is marked with a red dot.

Maize is a monoecious plant that produces a terminal male inflorescence (tassel) at the shoot apex and one or more lateral female inflorescences (ears) along the stalk axis (Bortiri and Hake, 2007). The timing of male and female flower maturation, or flowering time, greatly influences the potential grain yield at harvest time. Flowering time is therefore a very important trait to measure in a maize breeding program. To evaluate whether UAV-based BNDVI measurements contain information about male flowering time scored on the ground, we calculated Pearson’s correlation coefficients between the histogram PC values from each plot and tassel-appearance date. The most salient result in **Figure 6** is that slightly before the manually scored male flowering time (average for all genotypes), PC2 sharply increased and PC3 coordinately decreased. The biological basis of these coupled parameter changes is not known, but they could be useful indicators of maize flowering time.

### Yield: Correlations With Histogram Principal Components

**Figure 7:**
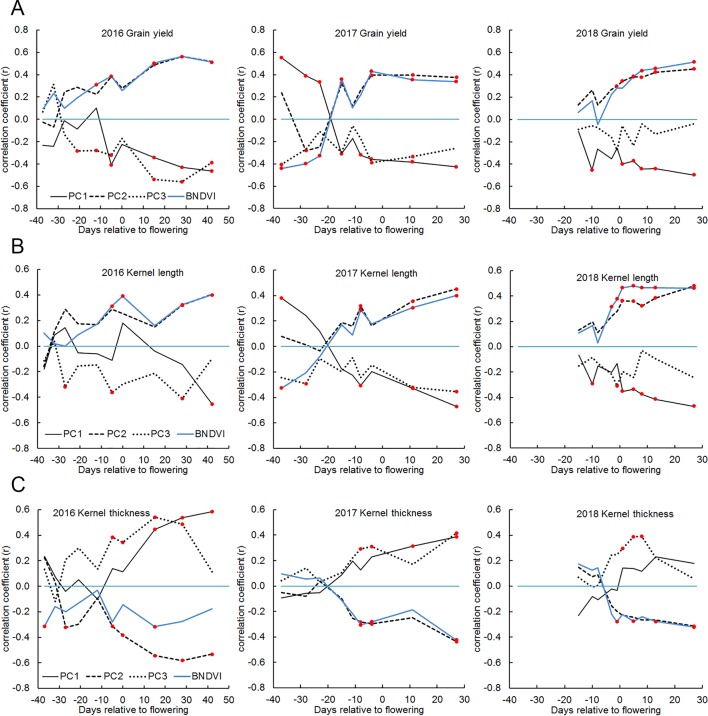
Correlations between yield, kernel dimensions and BNDVI histogram principal components in 2016, 2017, and 2018. **(A)** grain yield, **(B)** kernel length, and **(C)** kernel thickness. In each panel, the blue line indicates the correlation between the trait and mean BNDVI. The other lines represent correlation with the indicated principal component. Any correlation value that is significant at the P < 0.05 level is marked with a red dot.

The genotypes studied here varied more than two-fold with respect to grain yield measured at the end of the season (**Supplementary Table S1**). **Figure 7A** shows the correlation between the principal components and grain yield were as high as 0.6 after the flowering period. PC2 correlated positively and PC1 correlated negatively with grain yield. Because PC2 appeared to interpret the mean of the BNDVI distribution (**Figure 2C**), we tested the correlation between mean BNDVI and grain yield. Mean BNDVI and PC2 correlated with grain yield almost identically, indicating a greener canopy post-flowering is associated with a higher yield. PC1 negatively correlated with yield, indicating less visible ground (higher canopy coverage) after flowering is associated with a higher yield. PC3 negatively correlated with yield, indicating a less uniform canopy is associated with higher yield. Plant leaf greenness indicated by NDVI is an important trait, probably reflecting the ability to maintain robust photosynthesis needed for grain filling in the final growth stage (Zheng et al., 2009).

### Kernel Traits: Correlations With Histogram Principal Components

Grain yield is the product of kernel number and kernel weight. The latter is a function of kernel length, width, and thickness. The correlation between NDVI histograms and these separate components of yield was calculated. **Figure 7B** shows that kernel length displayed a correlation pattern similar to that displayed by grain yield (**Figure 7A**) while kernel thickness (distance each kernel occupies along the axis of the ear) displayed an inverse but otherwise similar correlation pattern (**Figure 7C**). The kernel width measurement returned by the analysis pipeline is probably related to and constrained by kernel row number, which is a highly heritable trait (Liu et al., 2016). This may explain why kernel width did not correlate well with BNDVI histogram principal components. Together, these results indicate that the features extracted from UAV-based images can be used to distinguish high-yielding genotypes from low yielding genotypes and that the higher yields are comprised of tall, thin kernels.

### Partial Least Squares Regression (PLS-R) Modeling

The analyses described thus far were designed to evaluate correlations at each sample date. As an alternative approach, we performed partial least squares regression (PLS-R) modeling to determine if the data contained in the BNDVI histograms obtained for each plot at all sample dates could predict the measured agronomic traits (flowering time, grain yield, and kernel dimensions). The PLS-R model was run 100 times, each time with 30% of the trials held out. The correlation coefficients obtained for each of the 100 runs were averaged and displayed in **Table 1**. The minimum number of factors needed to maintain predictive power of the model is also presented in **Table 1**. In some cases, especially for male and female flowering dates, a predictive relationship was discovered.

The results presented here are most useful if it can be assumed that the subjects (hybrid genotypes) are each unique and genetically unrelated. More caution would need to be exercised when interpreting the results if the genotypes belonged to a smaller number of classes characterized by shared portions of the genome. The potential source of misleading correlation to guard against is a familial relationship called population structure. It can be assessed by performing principal component analysis of genotype marker sets. Such an analysis of many of the genotypes used here is presented in **Supplementary Table S2**. The percent of genetic variation explained by the first four principal components of the genotype data was found to be 12.3, 6.0, 4.7, and 4.5. These genotype principal components did not correlate well with the BNDVI histogram principal components (**Supplementary Table S3**). Furthermore, PLS-R predictions of grain yield in 2016 and 2017 based on genotype principal components was 0.3, substantially lower than the predictions based on BNDVI histograms (**Table 1**). Thus, the BNDVI values derived from the UAV platform contain more predictive information than the genotype variables; population structure does not appear to be a significant confounder of the results and conclusions presented here.

## Discussion

Automated image acquisition followed by computational analysis promises to advance research on crops by producing better and new phenotype measurements (Perez-Sanz et al., 2017). Any approach begins with a decision about how to acquire images that will contain information about the process or trait of interest. Simplicity of design and operation is an important consideration when deciding on an image-acquisition platform (Kasampalis et al., 2018). The platform used here was based on a generic UAV and a compact digital camera mounted with its optical axis perpendicular to the ground. Open-source computer code developed by a photography community controlled the camera and the UAV was guided along routes created by free software running on a standard portable computer. The platform used standard global positioning systems (GPS) to navigate but not to tag the images. Neither GPS information nor ground reference points were necessary to construct full-field images from the highly overlapping component images. During a 5 minute flight session, the simple, low-cost UAV platform collected all the data needed to construct a full-field image comprising 50 plots with sub-centimeter resolution. Preliminary surveys of 1,000 two-row plots were completed in less than one hour.

There are some tradeoffs associated with the simple design and easy operation of the platform. The spectrally modified camera does not produce a standard NDVI measurement so the index values reported here can only be qualitatively compared with the results of other studies. To minimize the size of data files, the images were compressed and saved in jpeg format, which can distort color ratios and therefore affect vegetation indices (Verhoeven, 2010). Calibration against a color standard in the field was not performed so the effects of seasonal variation in sunlight quality were not removed from the data. While these issues would negatively affect the accuracy of an NDVI measurement, none of them affects the ability of the platform to quantitatively compare genotypes on a given date because images of each were acquired on clear days within minutes of each other. In fact, this platform produced an index that displayed a wide range and no saturation as the corn grew and matured, in contrast to a recent report of NDVI and its correlation with maize yield in different nitrogen conditions (Buchaillot et al., 2019).

It may be surprising that sampling two broad wavebands at 200,000 points within a plot across genotypes and time could produce metrics that correlated strongly with differences in flowering time, yield, and kernel thickness, but that is the central conclusion of this study. The correlations were reproducible across years. The predictive metrics derived from principal component analysis of BNDVI histograms have no traditional analog or manually-measured equivalent, but the correlations with yield were high enough to be useful in a selection process and higher than other researchers found using average NDVI as the metric (Spitkó et al., 2016; Buchaillot et al., 2019). The BNDVI histogram may be more useful for predictions than a simple plot average because multivariate analysis of histogram shape can de-emphasize uninformative values, for example those that may correspond to soil. The present results indicate that the metrics derived from the BNDVI histograms predict yield as well or better than genomic selection methodologies (Zhao et al., 2012). Our results support a recent suggestion to combine spectral information and genomic selection to improve breeding processes (Crossa et al., 2017). Other uses for mid-season information that reliably relates to yield include forecasting a year’s harvest and for inferring yield outcomes that were not accurately measured due to late-season storm damage.

Flowering time in relation to growing season has an important effect on yield. Traditionally, flowering time is measured by humans visually inspecting plots. Therefore, the method is prone to individual subjectivity. In 2018, two people independently scored flowering time of the plots studied in this project. The correlation between their two data sets was 0.82. Acquiring BNDVI data frequently during the flowering window with a simple UAV platform may more accurately and efficiently measure flowering time than human scorers. The feature in the data that appears to cause the correlation between NDVI and flowering is a transient reduction or dip, possibly due to the abrupt appearance of tassel material that is not green. In addition to being useful for determining the timing of tassel emergence, this feature in the data could serve as a proxy measurement of tassel size and structure (Gage et al., 2017), which may affect photosynthesis by shading the canopy (Duncan et al., 1967).

At a high level, yield is a function of canopy photosynthesis (source) and utilization of photosynthetic products especially in developing kernels (sink). Because a vegetation index measures the interaction between light and photosynthetic pigments, and may even correlate with photosynthetic capacity (Gamon et al., 1995), it is reasonable to consider that the platform used in this study measured source-related traits that affect yield. Lee and Tollenaar, (2007) argue that a slow rate of pigment loss in the latter stages of the season, a delay in senescence termed “stay green,” is associated with greater yield. The transcriptional and metabolic pathways associated with the stay-green phenomenon are beginning to be elucidated (Sekhon et al., 2019). The BNDVI histograms may have captured genotype-dependent differences in “stay green” that are associated with grain yield, resulting in the observed reproducible correlations with the histogram principal components. Performing more flights through the senescence period may improve yield predictions from BNDVI data by characterizing the senescence time course better. A more targeted or hypothesis-based analysis of the BNDVI histograms may capture the senescence time course better than the principal components approach taken here, resulting in better predictions.

The UAV platform described here collects data from which metrics that correlate well with flowering time, yield, and kernel dimensions can be computed. The bottleneck in the process is isolating the individual plots from the full field image. If future software development activities can automate this currently manual step, then the advantages of a simple aerial platform for measuring maize in a field can be put into full practice.

## Conclusion

The present study demonstrated that a UAV-based imaging platform assembled from consumer-grade components can generate measurements of a vegetation index that allowed the flowering time, yield, and kernel dimensions of maize hybrids to be compared. Rather than averaging the index values to summarize the plot, the frequency histograms of the values were decomposed into principal components. The three principal components that explained most of the variance were interpretable and predictive of tassel time, grain yield, kernel length, and kernel thickness. If a future software development effort could automate the step of isolating individual plots from the full-field image, the methods described here could allow essentially any research group to incorporate high-throughput predictive NGB imaging in their maize genetic research or breeding programs.

## Data Availability Statement

All datasets for this study are included in the manuscript/**Supplementary Files** or are available on request to the corresponding author.

## Author Contributions

ES, NL, SK, and GW conceived of the project. ES assembled the UAV platform and camera code. GW designed and performed the UAS experiments and data collection. NL and SK were responsible for establishing the plots. NM and GW performed the statistical analysis; GW and ES wrote the manuscript with feedback from the other authors.

## Funding

This work was supported by Iowa Corn and USDA-ARS.

## Conflict of Interest

The authors declare that the research was conducted in the absence of any commercial or financial relationships that could be construed as a potential conflict of interest.
