# Supplementary material for: Predicting Zea mays Flowering Time, Yield, and Kernel Dimensions by Analyzing Aerial Images
Source: Front Plant Sci. 2019 Oct 11;10:1251. doi: 10.3389/fpls.2019.01251 (PMC6797588; doi:10.3389/fpls.2019.01251)
Supplement: Supplementary file 1 [file DataSheet_1.pdf]

Supplementary Table S1.
Genotype principal component, mean BNDVI, yield and flowering time

Line	BNDVI	Grain yield (kg/plot)	Tassel date
**Year 2016**			
2369/PHZ51	0.49	15.05	7/26
B14A/C103	0.44	11.96	7/27
B37/H95	0.44	9.19	7/27
B73/MO17	0.48	12.11	7/26
B73/PHG35	0.52	16.49	7/27
B73/PHM49	0.48	23.62	7/27
B73/PHN82	0.49	23.01	7/27
B73/PHZ51	0.49	22.64	7/25
B73/TX205	0.50	21.14	7/28
CG60/LH162	0.45	14.78	7/19
GEMS-0150/PHZ51	0.45	16.53	7/26
GEMS-0162/PHZ51	0.45	14.86	7/27
LH132/LH51	0.47	22.34	7/25
LH74/PHN82	0.50	23.19	7/26
LH74/TX205	0.49	23.34	7/27
PB80/PHZ51	0.49	13.85	7/25
PHG39/PHG35	0.49	23.13	7/28
PHG39/PHM49	0.49	27.59	7/28
PHG39/PHN82	0.49	25.72	7/28
PHG39/TX205	0.49	17.85	7/31
PHW52/PHM49	0.50	25.05	7/26
PHW52/PHN82	0.50	23.44	7/26
PHW52/TX205	0.50	19.13	7/28
PHW52/TX205	0.50	27.60	7/28
TX714/PHZ51	0.48	17.75	7/26
WF9/MO17	0.43	17.34	7/26
**Year 2017**			
2369/3IIH6	0.52	25.42	7/26
2369/LH123HT	0.49	28.24	7/30
2369/PHN82	0.50	30.72	7/27
B14A/MO17	0.47	28.02	7/29
B37/H95	0.45	17.75	7/29
B73/MO17	0.47	28.16	7/28
B73/PHM49	0.47	29.64	7/28
B73/PHN82	0.49	25.61	7/27
B73/PHZ51	0.48	21.50	7/27
B73/TX205	0.50	24.19	7/30
GEMN-0260/3IIH6	0.50	27.36	7/25
GEMN-0268/3IIH6	0.46	26.44	7/27

LH132/LH51	0.47	30.11	7/28
LH198/PHN37	0.51	24.68	7/26
LH198/PHZ51	0.49	24.14	7/27
LH74/TX205	0.49	24.60	7/28
PB80/PHZ51	0.47	21.07	7/27
PHB47/PHZ51	0.49	18.29	7/26
PHG29/PHG47	0.44	21.60	7/25
PHG39/PHM49	0.51	26.14	7/29
PHW52/PHM49	0.51	24.58	7/28
PHW52/PHN82	0.48	29.06	7/27
W10004_0216/PHZ51	0.47	25.51	7/28
WF9/MO17	0.41	21.37	7/27
**Year 2018**			
2369/LH123HT	0.44	14.95	7/29
B14A/H95	0.43	6.32	7/22
B14A/MO17	0.39	10.87	7/25
B14A/OH43	0.43	8.35	7/27
B37/H95	0.43	11.46	7/28
B37/MO17	0.39	7.34	7/27
B37/OH43	0.40	5.83	7/27
B73/MO17	0.43	17.12	7/22
B73/PHM49	0.46	21.27	7/28
B73/PHN82	0.43	18.80	7/26
B73/TX779	0.43	11.66	7/26
CG119/CG108	0.47	15.24	7/29
F42/H95	0.41	10.57	7/26
F42/MO17	0.44	13.68	7/24
F42/OH43	0.42	16.70	7/25
LH195/PHZ51	0.44	15.54	7/27
LH216/LH195	0.42	10.93	7/22
LH74/PHN82	0.44	19.63	7/22
PHG39/PHN82	0.41	16.34	7/24
PHW52/PHM49	0.45	17.02	7/23
PHW52/PHN82	0.39	13.22	7/23
TX110/87916	0.43	16.50	7/23
TX714/TX779	0.42	19.25	7/26
TX777/LH195	0.41	13.84	7/27
TX779/LH195	0.37	6.79	7/23
Border	0.50	15.47	7/28

Supplementary Table S2 Genotype principal component, mean BNDVI, yield and flowering time

Line	PC1	PC2	PC3	PC4	BNDVI	Grain yield (kg/plot)	Tassel date
**Year 2016**							
2369/PHZ51	-52.53	9.31	12.33	-13.97	0.49	15.05	7/26
B14A/C103	8.85	-48.15	-13.70	-12.28	0.44	11.96	7/27
B37/H95	24.40	-22.75	3.57	-33.83	0.44	9.19	7/27
B73/Mo17	-29.91	-42.11	-32.81	58.22	0.48	12.11	7/26
B73/PHG35	-4.46	7.93	10.58	16.37	0.52	16.49	7/27
B73/PHM49	-5.95	17.32	14.56	19.22	0.48	23.62	7/27
B73/PHN82	8.55	26.35	29.88	53.28	0.49	23.01	7/27
B73/PHZ51	-83.74	13.35	2.69	-6.73	0.49	22.64	7/25
B73/TX205	-1.59	33.55	-72.89	20.10	0.50	21.14	7/28
GEMS-0150/PHZ51	-38.21	1.94	8.43	-21.75	0.45	16.53	7/26
GEMS-0162/PHZ51	-30.14	4.04	7.23	-20.80	0.45	14.86	7/27
LH132/LH51	1.72	-17.71	-6.54	8.22	0.47	22.34	7/25
LH74/PHN82	23.03	18.59	26.94	35.55	0.50	23.19	7/26
LH74/TX205	15.59	25.52	-60.74	5.69	0.49	23.34	7/27
PHB80/PHZ51	-32.46	5.18	7.69	-15.26	0.49	13.85	7/25
PHG39/PHG35	33.75	-3.89	16.32	-28.21	0.49	23.13	7/28
PHG39/PHN82	43.43	8.94	29.23	-4.10	0.49	25.72	7/28
PHG39/TX205	38.78	15.90	-42.27	-30.41	0.49	17.85	7/31
PHW52/PHM49	32.22	12.32	19.83	-18.84	0.50	25.05	7/26
PHW52/PHN82	37.14	20.05	31.38	17.39	0.50	23.44	7/26
PHW52/TX205	30.98	27.26	-58.21	-14.66	0.50	19.13	7/28
TX714/PHZ51	-43.37	6.87	7.11	-16.14	0.48	17.75	7/26
Wf9/Mo17	5.14	-85.25	-12.98	21.48	0.43	17.34	7/26
**Year 2017**							
2369/3IIH6	1.45	6.53	8.92	16.32	0.52	25.42	7/26
2369/LH123Ht	-3.52	-0.70	0.18	7.75	0.49	28.24	7/30
2369/PHN82	10.63	19.20	27.88	31.85	0.50	30.72	7/27
B37/H95	24.40	-22.75	3.57	-33.83	0.45	17.75	7/29
B73/Mo17	-29.91	-42.11	-32.81	58.22	0.47	28.16	7/28
B73/PHM49	-5.95	17.32	14.56	19.22	0.47	29.64	7/28
B73/PHN82	8.55	26.35	29.88	53.28	0.49	25.61	7/27
B73/PHZ51	-83.74	13.35	2.69	-6.73	0.48	21.50	7/27
B73/TX205	-1.59	33.55	-72.89	20.10	0.50	24.19	7/30
LH132/LH51	1.72	-17.71	-6.54	8.22	0.47	30.11	7/28
LH74/TX205	15.59	25.52	-60.74	5.69	0.49	24.60	7/28
PHB80/PHZ51	-32.46	5.18	7.69	-15.26	0.47	21.07	7/27
PHW52/PHM49	32.22	12.32	19.83	-18.84	0.51	24.58	7/28
PHW52/PHN82	37.14	20.05	31.38	17.39	0.48	29.06	7/27
W10004_0216/PHZ51	-50.47	6.24	6.39	-30.39	0.47	25.51	7/28
Wf9/Mo17	5.14	-85.25	-12.98	21.48	0.41	21.37	7/27

**Year 2018**							
2369/LH123Ht	-3.52	-0.70	0.18	7.75	0.44	14.49	7/29
B37/H95	24.40	-22.75	3.57	-33.83	0.43	11.46	7/28
B73/Mo17	-29.91	-42.11	-32.81	58.22	0.43	17.12	7/22
B73/PHM49	-5.95	17.32	14.56	19.22	0.46	21.27	7/28
B73/PHN82	8.55	26.35	29.88	53.28	0.43	18.80	7/26
LH74/PHN82	23.03	18.59	26.94	35.55	0.44	19.63	7/22
PHG39/PHN82	43.43	8.94	29.23	-4.10	0.41	16.34	7/24
PHW52/PHM49	32.22	12.32	19.83	-18.84	0.45	17.02	7/23
PHW52/PHN82	37.14	20.05	31.38	17.39	0.39	13.22	7/23

Supplementary Table S3. Correlation coefficients (r) and p values of defining the general lack of relationships between genotype PCs and the indicated traits in each of the three years.

		BNDVI				Grain yield				Tassel date			
	Year	PC1	PC2	PC3	PC4	PC1	PC2	PC3	PC4	PC1	PC2	PC3	PC4
r	2016	0.11	0.76	-0.01	0.19	0.33	0.52	0.18	0.23	0.53	0.20	-0.34	-0.26
p		0.62	0.00	0.95	0.38	0.12	0.01	0.41	0.30	0.00	0.19	0.02	0.08
r	2017	0.13	0.82	0.07	0.08	0.18	0.25	0.19	0.53	0.05	0.05	-0.43	-0.14
p		0.64	0.00	0.80	0.76	0.52	0.35	0.49	0.03	0.80	0.77	0.01	0.44
r	2018	-0.48	-0.03	-0.26	0.01	-0.34	0.40	0.14	0.56	-0.23	-0.03	-0.10	-0.33
p		0.19	0.94	0.51	0.98	0.38	0.28	0.73	0.12	0.36	0.89	0.70	0.17

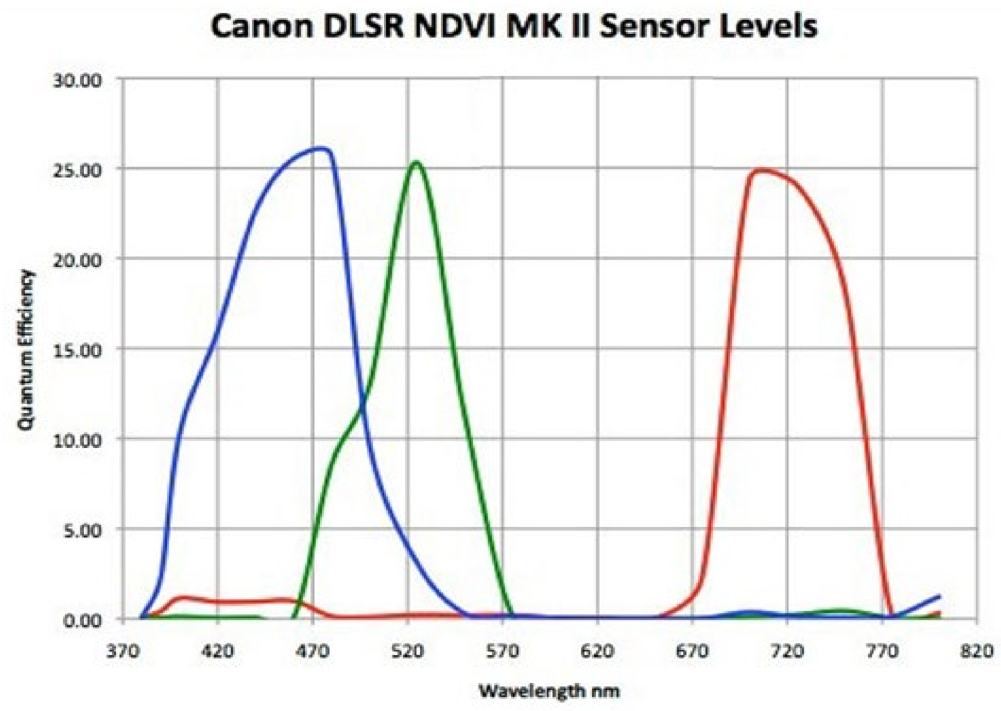

Figure S1. Spectral sensitivity of the blue, green, and infrared channels of the sensor after the camera was modified. The graph was provided by Llewellyn Data Processing.
